# Multi-omics approach to precision medicine for immune-mediated diseases

**DOI:** 10.1186/s41232-021-00173-8

**Published:** 2021-08-01

**Authors:** Mineto Ota, Keishi Fujio

**Affiliations:** 1 Department of Allergy and Rheumatology, Graduate School of Medicine, The University of Tokyo, Tokyo, 113-0033 Japan; 2 Department of Functional Genomics and Immunological Diseases, Graduate School of Medicine, The University of Tokyo, Tokyo, 113-0033 Japan

**Keywords:** Multi-omics analysis, Immune-mediated disease, Genome, Transcriptome, Expression quantitative trait loci, Polygenic risk score

## Abstract

Recent innovation in high-throughput sequencing technologies has drastically empowered the scientific research. Consequently, now, it is possible to capture comprehensive profiles of samples at multiple levels including genome, epigenome, and transcriptome at a time. Applying these kinds of rich information to clinical settings is of great social significance. For some traits such as cardiovascular diseases, attempts to apply omics datasets in clinical practice for the prediction of the disease risk have already shown promising results, although still under way for immune-mediated diseases. Multiple studies have tried to predict treatment response in immune-mediated diseases using genomic, transcriptomic, or clinical information, showing various possible indicators. For better prediction of treatment response or disease outcome in immune-mediated diseases, combining multi-layer information together may increase the power. In addition, in order to efficiently pick up meaningful information from the massive data, high-quality annotation of genomic functions is also crucial. In this review, we discuss the achievement so far and the future direction of multi-omics approach to immune-mediated diseases.

## Background

Immune-mediated diseases (IMDs) consist of a wide range of etiologies from autoimmune to autoinflammatory conditions [1]. Although a variety of therapeutic agents and regimens have been developed for each IMD, the treatment response varies from patient to patient. The heterogeneous pathogeneses of an IMD may be associated with different outcomes [2].

Recent advances in high-throughput sequencing technologies have enabled capturing comprehensive profiles of samples at multiple levels, referred to as omics analysis, for example, genomic, epigenomic, transcriptomic, and proteomic analyses. Multi-omics analysis refers to the collective analysis of omics data at multiple levels [3] and enables deep phenotyping of patients.

Personalized medicine, more recently referred to as precision medicine, aims to develop drugs and to optimize prescription of the appropriate drugs at the optimal dose and time [4, 5]. To achieve this aim, a deep understanding of the disease etiology, accurate and early diagnosis, appropriate stratification of patients based on disease phenotype, and prediction of the treatment response are required.

**Fig. 1:**
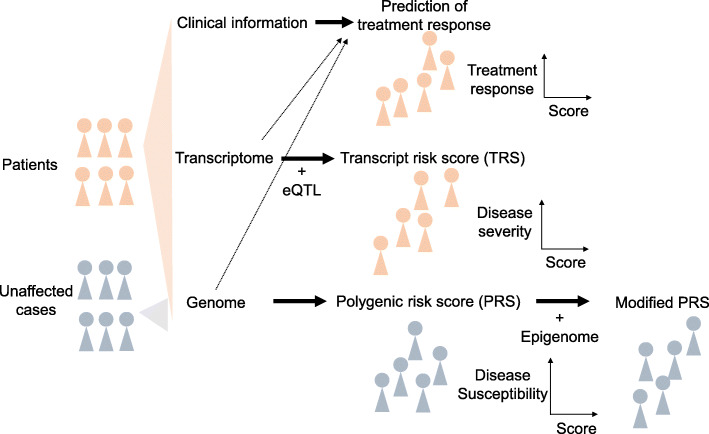
Present strategies for stratification of disease-affected and unaffected cases. How clinical information, transcriptome, and genome data can be used for predicting clinical outcomes or disease susceptibility.

Applying multi-omics information to precision medicine is a clinically attractive challenge. Although still in its infancy, there have been numerous efforts to apply omics data in clinical practice using various approaches (Fig. 1). Here, we review some of these approaches and discuss their potential future directions.

### Prediction of treatment response in rheumatoid arthritis

A growing number of drugs, from small molecule compounds to biologics, have been approved for IMDs so far [6]. In particular, many biological and non-biological disease-modifying anti-rheumatic drugs are widely used to treat rheumatoid arthritis (RA) [7, 8]; however, the treatment response varies from patient to patient, with only 30–40% of patients showing an adequate response to first-line biological disease-modifying anti-rheumatic drugs [9, 10]. Clinically, prediction of the treatment response before administering therapy to reduce the risk of side effects and the economic burden has gained great interest [10].

For this purpose, many studies have been conducted over the last 10 years (reviewed in detail elsewhere [11]). Briefly, patients serologically positive for rheumatoid factor or anti-citrullinated protein antibodies seem to show good responses to rituximab (RTX), an anti-CD20 monoclonal antibody [12, 13], to abatacept (CTLA4-Ig fusion protein) [14] and to tocilizumab (TCZ) [15], although serological positivity did not seem to be predictive of the response to TNF inhibitors [16]. In addition, a strong interferon (IFN) signature in serum was reported to predict a good response to TCZ [17] but a poor response to RTX [18].

Based on these findings, more specific and accurate markers for predicting treatment response have been investigated using omics data. Potential genetic variants associated with treatment response based on genomic data have been reported. For example, variants in the *PDE3A–SLCO1C1* [19], *CD84* [20], and *PTPRC* [21] loci and variants affecting the expression of *CD40* and *CD39* [22] were found to be associated with the response to TNF inhibitors; however, a limited number of those findings could be reproduced by a subsequent study [23], and thus further validation is required.

For fair evaluation of the usefulness of genomic data, one community-based assessment aimed to develop models predicting the response to TNF inhibitors in RA patients in 2016 [24]. In this open challenge, genomic data and clinical information from over 2000 patients were provided to 73 research groups to generate a prediction model using various approaches, including machine learning methods. Contrary to expectations, all of the groups failed to show a significant contribution of genetics to prediction accuracy. The highest performing model in this challenge greatly relied on clinical parameters, especially the 28-joint disease activity score at baseline [25]. Although this study reinforced the importance of clinical parameters for predicting treatment responses, clinical application of genetic data may still have a role in the prediction of drug responses, especially in larger sample sizes [26], and in combination with other omics levels such as transcriptomic data.

In contrast to genomic data, transcriptomic data reflect the variance acquired from environmental factors as well as from genetics. For instance, exposure to inflammation, cellular activation, or cellular composition can be reflected in transcriptomic data [27]. In some reports, transcriptomic data proved useful for patient stratification. In RA, synovial fibroblasts (SFs) play important roles in joint inflammation [28] and show a dynamic response to inflammation at the epigenomic and transcriptomic levels [29]. Lewis et al*.* classified RA patients into three distinct pathotypes (fibroblastic pauci-immune, macrophage-rich diffuse-myeloid, and lympho-myeloid pathotypes) based on gene expression in the synovium and assessed the treatment response associated with each pathotype using transcriptomic data [30]. They found that the pauci-immune pathotype was predictive of an inadequate response to TNF inhibitors [31]. In addition, Humby et al*.* recently conducted a biopsy-driven randomized controlled study (RTX vs. TCZ) and reported that “B-cell poor” patients (defined by RNA sequencing of SFs) showed a better response to TCZ than to RTX [32]. Although further validation is warranted, the transcriptomic data from diseased tissue might enable better stratification of IMD patients. Taken together, combining multi-level information, such as clinical information, serological information, and genomic/transcriptomic data, will improve the prediction of treatment responses in RA patients.

## Prediction of disease susceptibility using genomic data

Application of genomic data for precision medicine, especially for disease prevention, has gained wide interest in this era [33]. In particular, the polygenic risk score (PRS) has been applied to a variety of diseases, and some of the results have been promising. PRS is calculated by summing the effects of all common variants on disease onset to estimate the overall risk of developing a particular disease. It is especially useful for polygenic traits, in which small effects of numerous common variants contribute to disease onset. For instance, coronary artery disease (CAD) is a prevalent polygenic trait. In one study, individuals with a high PRS (8% of the population) had at least a threefold increased risk of developing CAD [34]. A clinical trial using the PRS for treatment intervention in CAD patients also had a promising outcome [35]. These results highlight the possibility of using genetic information to stratify high-risk patients, although predicting the risk of disease onset using only genomic data is still a challenge.

Generally, a larger sample size in genome-wide association studies (GWAS) is necessary for better prediction of the PRS, although the explained genetic variance of each trait also has an influence [36]. Among IMDs, relatively large-scaled GWAS for inflammatory bowel disease (IBD) have been performed [37, 38], and those datasets enabled promising disease risk prediction in the subsequent studies [34, 39]. In one of those studies, subjects with a PRS in the top 10% of distribution had a 2.43-fold increased disease susceptibility compared to the remaining 90% [34]. Although pre-disease interventions in IBD have not been established, some dietary habits have been associated with IBD onset [40, 41]. Thus, dietary intervention in high-risk cases could reduce the risk of disease onset.

In clinical practice in IMDs, correct diagnosis at the first outpatient visit is important. One interesting study evaluated the value of genetic data combined with clinical examination for accurate diagnosis of inflammatory arthritis [42]. In that study, based on retrospective data, the authors indicated that calculating disease risk using genetic information significantly improved the accuracy of the initial diagnosis of arthritis. As another example, Zhao et al*.* reported that the DNA methylation level at the *IFI44L* locus distinguished SLE patients from healthy controls, RA patients, and Sjögren’s syndrome patients, suggesting its potential as a diagnostic marker for SLE [43]. Those studies support the utility of genomic and epigenomic data for improving clinical practice in rheumatology, although further validation by prospective studies in real clinical settings is required.

## Attempts to improve the performance of the PRS

Although clinical application of the PRS to classify increased risks of certain traits has become realistic, PRSs developed by GWAS for a specific population tended to underperform when tested in a different population [44, 45]. As most large-scale GWAS have been performed in European populations, this might lead to health disparities among populations [45].

Some attempts to overcome this issue have already been reported. Amariuta et al*.* reported that the trans-ethnic portability of the PRS was significantly improved by prioritizing variants with regulatory annotation which was constructed based on epigenomic data [46]. Another group reported that calculating the PRS based on variants discovered among diverse populations improved the trans-ethnic portability of the PRS [47]. Although conducting large-scale GWAS in diverse populations is also an important direction [48], combination with other omics data, such as epigenomic data, would improve the performance of the PRS in the clinical setting.

## Application of epigenomic data for multi-omics analysis

As exemplified above, epigenomic data is a quite valuable resource for multi-omics analysis. Recently, Encyclopedia of DNA elements (ENCODE) Project has released their phase III data, which consists of wide variety of epigenomic annotations from 5992 new experimental datasets [49]. Together with Roadmap Epigenomics data [50], these large-scaled epigenome datasets inform cis-regulatory elements of variety of cell types. As epigenomic data is not influenced by linkage disequilibrium or allele frequencies, these data can be utilized for prioritization of disease-associated genetic variants in the context of precision medicine.

## Application of expression quantitative trait loci (eQTL) data for precision medicine

eQTL analysis is used to identify the association between genetic variants and gene expression. During the past 10 years, large consortia including Geuvadis [51], GTEx [52], DICE [53], and eQTLGen [54] performed eQTL analyses in various tissues and cell types, using large sample sizes, and reported the effects of genetic variants on gene expression. The resulting datasets are quite useful for estimating the effects of disease-associated variants. Generally, majority of non-coding disease-associated genetic variants are assumed to modulate the expression of genes that play roles in disease pathogenesis. Thus, it is reasonable to integrate GWAS results with eQTL data and estimate gene-level associations with diseases to reduce the multiple testing burden and facilitate biological interpretation. This approach, referred to as transcriptome-wide association studies (TWAS), has garnered great interest, and many such studies have been performed [55–57].

Integration of eQTL and GWAS datasets can be applied for patient-level estimation of disease risk. Marigorta et al*.* developed a transcriptional risk score (TRS) based on the gene expression data in ileal mucosal samples from Crohn’s disease patients, risk variants of Crohn’s disease determined by GWAS, and the eQTL effects of these variants [58]. The TRS outperformed genetic risk scores in terms of not only distinguishing Crohn’s disease from healthy samples but also identifying patients who will progress to complicated disease [58]. Although further validation in other centers is warranted, that study raises the possibility of treating gene expression data derived from biopsy specimens as an index for patient classification, in combination with GWAS and eQTL data.

To enhance understanding of the functions of IMD-associated genetic variants, their functions in immune cells should be evaluated. Recently, we constructed an eQTL atlas based on 28 types of immune cells (ImmuNexUT; Immune Cell Gene Expression Atlas from the University of Tokyo) [27]. Our atlas showed enrichment of IMD-associated genetic variants in immune cell eQTLs and identified a number of IMD-associated genes and cell types. This information could be used to prioritize disease-relevant cell types and genes and subsequently stratify IMD patients in the future. In addition, information on genetic variants associated with heterogeneity within a disease is limited so far. In a GWAS of IBD, few genetic variants were associated with disease prognosis [59]. The difficulty of clinically defining heterogeneity within a disease and the small number of patients with a rare disease entity make it a challenge to identify disease heterogeneity-associated variants. Using ImmuNexUT, we identified immune cell eQTL variants that show heterogeneity in effect sizes in an inflammatory context-dependent manner. These context-dependent eGenes (genes possessing context-dependent eQTL variants) are enriched in genes induced by inflammation or vaccination, indicating their roles in diversifying an individual’s response to inflammation. We surmise that these context-dependent eQTL variants are candidates for IMD heterogeneity and will aid prioritization of variants and stratification of IMD patients.

## Conclusion

For predicting disease susceptibility, disease severity, and treatment response, multi-omics data may play an important role in clinical practice in the near future. So far, some attempts at patient stratification have been performed using genomic, epigenomic, transcriptomic, and clinical data; however, most of those studies were based on information from a single level. Combination of multi-level information will improve the prediction of these outcomes. Construction of large-scale patient cohorts with high-quality clinical data (e.g., treatment response, clinical prognosis, and genomic, transcriptomic, and epigenomic data, preferably from diseased tissues) and refined analytic approaches to handle these data would contribute to a better understanding of IMD biology and accelerate precision medicine in IMD patients.

## Data Availability

Not applicable.

## Abbreviations

IMD: Immune-mediated disease
RA: Rheumatoid arthritis
RTX: Rituximab
TCZ: Tocilizumab
IFN: Interferon
PRS: Polygenic risk score
SF: Synovial fibroblast
CAD: Coronary artery disease
GWAS: Genome-wide association study
IBD: Inflammatory bowel disease
eQTL: Expression quantitative trait loci
TWAS: Transcriptome-wide association study
TRS: Transcriptional risk score
ImmuNexUT: Immune Cell Gene Expression Atlas from the University of Tokyo
